# Lipoprotein profiles of fat distribution and its association with insulin sensitivity

**DOI:** 10.3389/fendo.2022.978745

**Published:** 2022-10-25

**Authors:** Dongmei Wei, Vannina González Marrachelli, Jesus D. Melgarejo, Chia-Te Liao, Stefan Janssens, Peter Verhamme, Thomas Vanassche, Lucas Van Aelst, Daniel Monleon, Josep Redón, Zhen-Yu Zhang

**Affiliations:** ^1^ Studies Coordinating Centre, Research Unit Hypertension and Cardiovascular Epidemiology, Department of Cardiovascular Sciences, University of Leuven, Leuven, Belgium; ^2^ Department of Physiology, Faculty of Medicine, University of Valencia, Valencia, Spain; ^3^ INCLIVA Research Institute, University of Valencia, Valencia, Spain; ^4^ Division of Cardiology, University Hospitals Leuven, Leuven, Belgium; ^5^ Department of Cardiovascular Sciences, University of Leuven, Leuven, Belgium; ^6^ Department of Pathology, University of Valencia, Valencia, Spain

**Keywords:** Obesity, waist-to-hip ratio, lipoprotein, insulin resistance, cardiovascular risk

## Abstract

**Background:**

Fat deposition is associated with adverse outcomes. Waist-to-hip (WHR) ratio is a simple feasible index to assess fat distribution. Lipoprotein particle composition in relation to WHR and to what extent their association is mediated by insulin sensitivity are less investigated.

**Methods:**

In 504 randomly recruited Flemish (mean age: 48.9 years; women: 51.6%), we analyzed the lipoprotein particle constitutions using nuclear magnetic resonance spectroscopy. WHR obesity described a WHR of ≥ 0.85 for women or 0.9 for men. Insulin sensitivity was evaluated by the homeostasis model assessment-estimated insulin resistance (HOMA-IR). SCORE-2 risk algorithm was applied to estimate 10-year cardiovascular risk. Statistical methods included multivariable-adjusted linear regression analysis, logistic regression analysis, and mediation analysis.

**Results:**

The prevalence of WHR obesity was 54.6%, approximately 3 times of BMI-determined obesity (19.1%). Individuals with WHR obesity had significantly higher metabolic complications, such as hypertension (57.1%), dyslipidemia (61.8%), and insulin resistance (14.2%). WHR and WHR obesity were positively associated with total very-low-density lipoprotein (VLDL) particle concentration, remnant cholesterol, and triglycerides, but were negatively associated with VLDL particle size (P ≤ 0.027), independent of body mass index and other covariates. WHR was inversely associated with total high-density lipoprotein (HDL) particle concentration, whereas WHR obesity was inversely associated with HDL cholesterol (P ≤ 0.039). Neither WHR nor WHR obesity was associated with the concentration of total low-density lipoprotein (LDL) particles, LDL particle size, and LDL cholesterol (P ≥ 0.089). In the mediation analysis, insulin sensitivity significantly mediated the effect of WHR on total VLDL particle concentration (mediation percentage: 37.0%), remnant cholesterol (47.7%), and HDL cholesterol (41.1%). Individuals with WHR obesity were at increased cardiovascular risk, regardless of LDL cholesterol (P ≤0.028). In WHR obesity, higher total VLDL particle concent36ration and remnant cholesterol, and lower HDL cholesterol were associated with an increased cardiovascular risk (P≤ 0.002).

**Conclusions:**

Upper-body fat deposition was independently associated with an unfavorable lipoprotein profile, and insulin sensitivity significantly mediated this association. LDL cholesterol might underestimate lipid abnormality for people with upper-body obesity and lowering VLDL particles and remnant cholesterol might potentially reduce the residual cardiovascular risk.

## Introduction

The prevalence of obesity, one of the top threats to global public health, has nearly tripled from 1975 to 2016 (1). Obesity strikingly increases the risk of various noncommunicable diseases, including type 2 diabetes mellitus, cardiovascular disease, and mortality (2). It is extensively acknowledged that obesity is generally accompanied by metabolic comorbidities, including insulin resistance and dyslipidemia. Apart from excessive overall fat accumulation, adipose tissue distribution, especially abdominal fat deposition, is strongly associated with an increased risk of all-cause mortality, cardiovascular events, and insulin resistance (3–7). Since body mass index (BMI) is prevailingly used to assess overall fat accumulation and to associate with cardiovascular disease, most studies on lipid associations investigated BMI as a surrogate of obesity (8–12). Waist-to-hip ratio (WHR) is a simple and cheap indicator of fat distribution, and is relatively less correlated with BMI, compared to waist circumference (13). The positive association between WHR and cardiovascular risk has been suggested by large prospective studies (13, 14). WHR has been repetitively associated with conventional lipid parameters, such as low-density lipoprotein (LDL) cholesterol, whereas the association between WHR and more detailed lipoprotein particle compositions is less investigated (15–18). Linking WHR to complex lipoprotein composition might promote the utilization of WHR, provide more information for lipid-lowering options, and recognize the residual cardiovascular risk for individuals with abdominal obesity.

Insulin resistance, a prevalent metabolic complication of obesity, has a profound impact on lipid metabolism. Numerous epidemiological studies have investigated the association of insulin resistance with the anthropometric indices of obesity and the cholesterol contents or particles constitutions of different lipoproteins (19, 20). However, it remains unclear to what extent insulin sensitivity intermediates the effect of fat distribution on lipoproteins. Determining the effect mediated by insulin sensitivity could further uncover the mechanism of lipid abnormality in individuals with upper-body obesity and potentially facilitate the development of effective intervention strategies. Thus, a simultaneous investigation of insulin sensitivity, WHR, and lipoprotein particle constitutions is necessary to quantify the mediated effect of insulin sensitivity using mediation analysis.

Therefore, this study evaluated the association between lipoprotein particle composition and WHR, and further investigated the mediator role of insulin sensitivity in the general population. We additionally associated the lipoprotein profile of WHR with the estimated cardiovascular risk score to sharpen the understanding of the lipoprotein profile.

## Materials and methods

### Participants

All participants were from the Flemish Study on Environment, Genes and Health Outcomes (FLEMENGHO), a large prospective study that included 3343 individuals from the Flemish region from 1985 until 2004 with a participation rate of 78% at enrollment (21). The present study was approved by the University of Leuven Ethics Committee and written informed consent was obtained from all participants prior to study participation. All procedures were in accordance with the ethical principles of the Declaration of Helsinki. Participants who underwent lipoprotein profiling using nuclear magnetic resonance (NMR) spectroscopy were eligible for this study. Of 593 eligible participants, 89 participants who received lipid-lowering drugs were excluded due to the potential influence of lipid-lowering drugs on lipoprotein constitutions. Thus, this study eventually included 504 participants.

### Anthropometric measurements

The measurement of weight and height was performed with standardized equipment and procedures. BMI was calculated by body weight (kg) divided by the square of height (m^2^). Waist circumference was measured at the midway between the lower ribs and the top of the iliac crest to the nearest 0.1 cm. Hip circumference was measured at the widest portion of the buttocks. Waist-to-hip ratio (WHR) indicated the ratio of waist circumference and hip circumference. The measurement was completed when participants were in the upright position, and waist circumference was measured at the end of expiration. BMI obesity was defined as a BMI of ≥ 30 kg/m^2^. WHR obesity described a WHR of ≥ 0.85 for women or 0.9 for men (22).

### Nuclear magnetic resonance (NMR) spectroscopy measured lipoproteins

The fasting venous blood samples were centrifuged after collection, and the obtained plasma samples were preserved under -80°C until further analysis. The lipoprotein profiling was measured by 2D diffusion-ordered ^1^HNMR spectroscopy (DOSY) at INCLIVA Molecular and Metabolomics Image Lab, Valencia, Spain (23). The sample preparation and the protocol of analysis were detailed elsewhere (24). After being transferred into 5 mm NMR tubes, the prepared samples were randomized, and kept at 4°C until measurement (mean time until measurement: 6 hours). Samples were then inserted in the magnet, warmed to 37°C and ^1^H NMR spectra were acquired in a Bruker Avance III 600 spectrometer with an operating frequency at 600.20 MHz. The double stimulated echo pulse program with bipolar gradient pulses and a longitudinal eddy current delay was used. The obtained lipoproteins signals were deconvoluted and analyzed separately based on the diffusion properties and its associated NMR size into main fractions: very-low-density lipoprotein (VLDL) (38.6-81.9 nm), low-density lipoprotein (LDL) (18.9-26.5 nm), high-density lipoprotein (HDL) (7.8-11.5 nm) as detailed elsewhere (24). Particle size was then calculated based on its diffusion properties and each fraction was further divided into large, medium, and small particle subclass according to their particle diameters. The average lipoprotein particle size was calculated by averaging the NMR area of each fraction by its associated size.

### Other measurements

The venous blood samples were collected from August 2005 to March 2015 and obtained after at least 8 hours of fasting for the conventional lipid profile measurements, plasma glucose, and insulin. The conventional lipid measures included total cholesterol, LDL cholesterol, HDL cholesterol, and triglycerides, which were measured by using automated methods in certified laboratories. Specifically, since remnant cholesterol, indicative of the cholesterol content of the triglyceride-rich lipoproteins, has been associated with cardiovascular risk recently, it was also included as a conventional lipid parameter in this study. Remnant cholesterol was estimated by total cholesterol minus LDL cholesterol minus HDL cholesterol. The homeostasis model assessment-estimated insulin resistance (HOMA-IR) was calculated by multiplying plasma glucose (mmol/L) by insulin (uIU/mL), divided by 22.5. Diabetes mellitus was defined as fasting blood glucose of ≥126 mg/dL or receiving antidiabetic drugs. Hypertension was an office blood pressure of ≥140 mmHg systolic or ≥90 mmHg diastolic, or the use of antihypertensive drugs. Dyslipidemia was defined as LDL-cholesterol ≥ 3.36 mmol/L (130 mg/dL) or total cholesterol ≥ 5.17 mmol/L (200 mg/dL) or HDL-cholesterol ≤ 1.29 mmol/l (50 mg/dL) in women and 1.03 mmol/l (40 mg/dL) in men or fasting triglycerides ≥ 1.70 mmol/L (150 mg/dL) according to the criteria of Adult Treatment Panel III (25). Insulin resistance was defined as HOMA-IR of ≥ 2.5. Glomerular filtration rate was estimated using the chronic kidney disease epidemiology collaboration creatinine equation (26). The 10-year fatal and non-fatal cardiovascular risk (%) was estimated with SCORE2 risk prediction algorithms based on sex, age, smoking status, total and HDL cholesterol, and systolic blood pressure (27).

### Statistical analyses

Data analyses were performed with SAS software, version 9.4 (SAS Institute, Cary, NC, USA). Means and proportions were compared by t-test and Wilcoxon test as appropriate. Statistical significance was a two-sided P value of 0.05. The concentration of lipoprotein particles was normalized by the transformation of the logarithm to base 2. The correlation between the NMR-measured lipoprotein particles and conventional lipid variables was assessed by Spearman’s rank correlation. Multivariable-adjusted linear regression models were applied to assess the association of continuous WHR with lipid parameters. The following variables were considered as covariates: sex, age, current smoking, current alcohol assumption, and blood glucose. These covariates were considered based on their clinical relevance, the association with obesity, and literature (8, 28, 29). The collinearity of linear models was examined. In categorical analysis, the association of WHR obesity with lipid parameters was assessed using multivariable-adjusted logistic regression models with the adjustment of the same covariates.

The mediation analysis was performed by the following steps: 1) To examine whether insulin sensitivity was a potential mediator, the associations between insulin sensitivity, WHR, and lipid parameters were examined. The association of WHR with HOMA-IR indicated the effect (a) of WHR on the mediator. The association of HOMA-IR with a lipid parameter denoted the effect (b) of mediator on lipid parameter. 2) The association of WHR with a lipid parameter was the total effect (c). 3) Whether insulin sensitivity intermediated the association between WHR and lipid parameters was analyzed by the mediation model using HOMA-IR as a mediator. The total effect (c) of WHR on a parameter comprised direct effect and indirect effect. The indirect effect (a*b) represented the mediated effect of WHR on a lipid parameter through insulin sensitivity (WHR → HOMA-IR → a lipid parameter). The direct effect (c’) referred to the remaining effect of WHR on lipid parameters, not intermediating *via* insulin sensitivity (WHR → other paths → a lipid parameter). The proportion of indirect effect to total effect was the mediation percentage of HOMA-IR. The mediation analysis was performed in SAS with the PROC CAUSALMED Procedure. All associations in mediation models were expressed as β coefficients and were adjusted for sex and age.

## Results

### Participant characteristics

**Table 1** shows the characteristics of 504 participants. The age (SD) averaged 48.9 ( ± 15.4) years, and 260 (51.6%) were female. Of 504 participants, 275 (54.6%) had WHR obesity, approximately 3 times of BMI obesity (96, 19.1%). Individuals with WHR obesity had significantly higher cardiometabolic complications: higher prevalence of hypertension (57.1% vs. 27.1%), dyslipidemia (61.8% vs. 44.1%), insulin resistance (14.2% vs. 4.8%), compared to those without WHR obesity. The clinical risk factors in individuals with WHR obesity presented an unfavorable trend as well: older, higher blood pressure, blood glucose, insulin, HOMA-IR, and 10-year cardiovascular risk score (P <0.0001).

**Table 1:** Participant characteristics.

Characteristics	All	Normal WHR	WHR obesity	P
	(n = 504)	(n = 229)	(n = 275)	
Number with characteristic (%)				
Female	260 (51.6)	144 (62.9)	116 (42.2)	<0.0001
Current Smoking	76 (15.1)	38 (16.6)	38 (13.8)	0.45
Current alcohol	369 (73.2)	174 (76.0)	195 (70.9)	0.23
BMI ≥ 30 kg/m^2^	96 (19.1)	13 (5.7)	83 (30.2)	<0.0001
Diabetes mellitus	8 (1.6)	1 (0.4)	7 (2.6)	0.077
Cardiovascular diseases	29 (5.8)	11 (4.8)	18 (6.6)	0.45
Hypertension	219 (43.5)	62 (27.1)	157 (57.1)	<0.0001
Treatment of hypertension	87 (17.3)	27 (11.8)	60 (21.8)	0.003
Dyslipidemia	271 (53.8)	101 (44.1)	170 (61.8)	<0.0001
Insulin resistance	50 (9.9)	11 (4.8)	39 (14.2)	0.0005
Mean ± SD or median (IQR) of characteristic				
Age, years	48.9 ± 15.4	43.2 ± 15.7	53.6 ± 13.5	<0.0001
BMI, kg/m^2^	26.1 ± 4.6	23.7 ± 3.4	28.0 ± 4.5	<0.0001
WHR	0.88 ± 0.08	0.81 ± 0.05	0.94 ± 0.06	<0.0001
Waist circumference, cm	90.7 ± 13.0	81.5 ± 8.8	98.4 ± 10.7	<0.0001
Hip circumference, cm	102.9 ± 8.8	100.6 ± 8.1	104.9 ± 8.9	<0.0001
Systolic blood pressure, mmHg	129.9 ± 17.2	125.7 ± 17.4	133.5 ± 16.1	<0.0001
Diastolic blood pressure, mmHg	81.9 ± 9.8	78.7 ± 9.3	84.5 ± 9.4	<0.0001
Total cholesterol, mmol/L	4.94 ± 0.90	4.79 ± 0.85	5.07 ± 0.92	0.0003
LDL cholesterol, mmol/L	2.92 ± 0.78	2.73 ± 0.72	3.07 ± 0.79	<0.0001
HDL cholesterol, mmol/L	1.52 ± 0.42	1.65 ± 0.41	1.41 ± 0.39	<0.0001
Remnant cholesterol, mmol/L	0.50 ± 0.27	0.41 ± 0.18	0.59 ± 0.40	<0.0001
Triglycerides, mmol/L	1.09 ± 0.61	0.89 ± 0.39	1.26 ± 0.71	<0.0001
Non-HDL, mmol/L	3.42 ± 0.90	3.14 ± 0.79	3.67 ± 0.92	<0.0001
Blood glucose, mmol/L	5.10 (3.40-8.10)	4.00 (2.80-6.00)	6.10 (4.00-9.00)	0.21
Insulin, uIU/mL	1.03 (0.67-1.69)	0.82 (0.56-1.20)	1.28 (0.82-1.95)	<0.0001
HOMA-IR	2.22 (0.88-4.79)	1.19 (0.44-2.95)	3.57 (1.60-5.97)	<0.0001
10-years SCORE2, %	0.81 (0.71-0.95)	0.77 (0.68-0.89)	0.85 (0.73-0.98)	<0.0001
eGFR, ml/min/1.73m^2^	95.3 ± 16.7	100.8 ± 16.0	90.8 ± 16.0	<0.0001

WHR obesity was defined as WHR ≥ 0.85 for women, 0.9 for men. BMI, body mass index; eGFR, estimated glomerular filtration rate; HDL, high-density lipoprotein; HOMA-IR, homeostatic model assessment for insulin resistance; IQR, interquartile range; LDL, low-density lipoprotein; SD, standard deviation; WHR, waist-to-hip ratio.

### Lipid parameters in individuals with WHR obesity

For conventional lipid parameters, individuals with WHR obesity had elevated concentrations of total cholesterol, LDL cholesterol, remnant cholesterol, and triglycerides, but lower HDL cholesterol (**Table 1**). **Table 2** shows NMR-measured lipoprotein constitutions in individuals with and without WHR obesity. Compared to the normal WHR group, individuals with WHR obesity had significantly higher total VLDL particle concentrations (P <0.0001), and lower total HDL particle concentrations (P=0.0003). However, neither total LDL particle concentration nor any LDL particle subclass concentration (large, medium, and small fraction) showed a particular trend across the BMI categories (P ≥ 0.054). The correlation between the NMR spectrometry measured lipoprotein particle parameters and conventional lipid parameters is shown in **Table S1** in the supplementary information. Remnant cholesterol and triglycerides from the conventional lipid measurement were highly correlated with VLDL particle concentration (r: 0.914 and 0.913, respectively). LDL particle concentration was proportionally correlated with LDL cholesterol and total cholesterol (r: 0.723 and 0.776, respectively). HDL cholesterol was positively correlated with HDL particle concentration (r: 0.608), whereas it was negatively correlated with VLDL particle concentration (r: -0.504).

**Table 2:** NMR spectrometry-measured lipoprotein particle concentration and size.

	Normal WHR	WHR obesity	P
	(n = 229)	(n = 275)	
Lipoprotein particle concentration, nmol/L			
VLDL particles			
Total	24.63 (17.32-33.61)	36.91 (25.38-55.44)	<0.0001
Large	0.74 (0.53-1.01)	0.93 (0.73-1.39)	<0.0001
Medium	3.16 (2.20-4.36)	4.76 (3.18-7.50)	<0.0001
Small	20.75 (14.69-27.64)	30.99 (21.51-46.78)	<0.0001
Cholesterol	3.62 (0.25-8.34)	9.09 (3.83-16.96)	<0.0001
TG	39.28 (29.22-50.85)	54.08 (40.01-79.08)	<0.0001
IDL particles			
Cholesterol	2.90 (0.68-5.36)	3.96 (1.76-6.90)	0.0003
TG	3.54 (1.37-5.66)	4.43 (2.52-7.17)	0.0004
LDL particles			
Total	507.14 (358.50-664.78)	544.30 (402.66-705.84)	0.088
Large	77.34 (54.96-103.99)	83.63 (57.74-109.31)	0.070
Medium	173.40 (123.13-225.07)	175.44 (135.51-234.29)	0.240
Small	257.05 (190.16-340.95)	279.78 (206.74-365.00)	0.054
Cholesterol	68.88 (50.13-89.63)	75.14 (52.68-98.81)	0.061
TG	9.49 (4.30-14.58)	9.68 (5.29-14.49)	0.50
HDL particles			
Total	22.76 (19.01-27.99)	19.59 (15.17-26.03)	0.0003
Large	0.23 (0.15-0.39)	0.26 (0.15-0.42)	0.48
Medium	7.24 (5.45-8.79)	5.56 (4.11-7.63)	<0.0001
Small	15.36 (12.47-18.78)	14.11 (10.52-18.07)	0.009
Cholesterol	51.63 (43.05-59.95)	42.82 (34.40-53.30)	<0.0001
TG	4.28 (0.54-8.18)	6.19 (2.27-10.80)	<0.0001
Average particle size, nm			<0.0001
VLDL particles	42.01 (41.70-42.38)	41.81 (41.57-42.15)	0.0001
LDL particles	21.08 (21.02-21.14)	21.09 (21.01-21.17)	0.70
HDL particles	8.24 (8.22-8.25)	8.23 (8.20-8.25)	<0.0001

WHR obesity was defined as WHR ≥ 0.85 for women, 0.9 for men. The lipid particle concentrations and average sizes are expressed as median (interquartile range). HDL, high-density lipoprotein; IDL, intermediate-density lipoprotein; LDL, low-density lipoprotein; TG, triglycerides; VLDL, very low-density lipoprotein, WHR, waist-to-hip ratio.

### The association of WHR with lipids parameters

**Table 3** displays the adjusted linear association of WHR with the lipoprotein constitutions with adjustment of sex, age, current smoking, current alcohol assumption, and blood glucose (model 1). Total VLDL particle concentration and the concentration of VLDL particle subclasses, cholesterol, and triglycerides contents were proportionally increased with WHR (P ≤ 0.0001, **Table 3**), independent of covariables. With the additional adjustment of BMI (model 2), WHR was still positively associated with the concentration of total VLDL particle and VLDL particle subclasses (P ≤ 0.047). Total HDL particle concentration, medium and small HDL particle concentration, and cholesterol and triglycerides contents were inversely associated with WHR (P ≤ 0.011). The association of WHR with the concentration of total and medium HDL particle remained significant after further adjusting for BMI. High WHR was associated with smaller VLDL particle size, independent of BMI (P = 0.0005). Notably, higher WHR was not associated with LDL particle concentration, cholesterol and triglycerides contents, and LDL particle size (P ≥ 0.56). Along the same line, the conventional lipid parameters presented similar associations with WHR, as indicated by elevated remnant cholesterol and triglycerides, mainly derived from VLDL particles (P ≤ 0.027). The associations of WHR with HDL-cholesterol and LDL cholesterol were disappeared when additionally adjusting for BMI (P ≥ 0.14).

**Table 3:** Linear association of WHR with lipid parameters.

	WHR			
	Model 1: coefficient (95% CI)	P	Model 2: coefficient (95% CI)	P
Lipoprotein particle concentration				
VLDL particles				
Total	0.024 (0.018 to 0.030)	<0.0001	0.010 (0.004 to 0.016)	0.001
Large	0.021 (0.014 to 0.029)	<0.0001	0.0071 (0.0001 to 0.0141)	0.047
Medium	0.022 (0.016 to 0.028)	<0.0001	0.008 (0.003 to 0.014)	0.005
Small	0.024 (0.018 to 0.031)	<0.0001	0.010 (0.004 to 0.016)	0.001
Cholesterol	0.005 (0.003 to 0.007)	<0.0001	0.0012 (-0.0005 to 0.0029)	0.16
Triglyceride	0.028 (0.021 to 0.036)	<0.0001	0.012 (0.005 to 0.019)	0.0009
IDL particles				
Cholesterol	0.002 (-0.0003 to 0.003)	0.10	-0.0001 (-0.0017 to 0.0016)	0.94
Triglyceride	0.004 (0.001 to 0.007)	0.012	0.001 (-0.002 to 0.004)	0.45
LDL particles				
Total	0.001 (-0.007 to 0.009)	0.78	0.001 (-0.006 to 0.008)	0.80
Large	0.002 (-0.006 to 0.010)	0.56	0.003 (-0.004 to 0.010)	0.41
Medium	-0.002 (-0.010 to 0.006)	0.60	-0.0004 (-0.0072 to 0.0064)	0.91
Small	0.002 (-0.006 to 0.010)	0.58	0.001 (-0.006 to 0.008)	0.76
Cholesterol	-0.001 (-0.008 to 0.006)	0.83	0.0004 (-0.0055 to 0.0063)	0.90
Triglyceride	0.001 (-0.002 to 0.003)	0.57	0.0005 (-0.0015 to 0.0024)	0.64
HDL particles				
Total	-0.011 (-0.018 to -0.004)	0.003	-0.006 (-0.012 to 0.000)	0.039
Large	-0.001 (-0.006 to 0.004)	0.72	-0.0004 (-0.0047 to 0.0039)	0.85
Medium	-0.013 (-0.020 to -0.007)	<0.0001	-0.007 (-0.013 to -0.002)	0.013
Small	-0.009 (-0.016 to -0.002)	0.011	-0.0057 (-0.0116 to 0.0003)	0.063
Cholesterol	-0.034 (-0.045 to -0.023)	<0.0001	-0.016 (-0.026 to -0.006)	0.002
Triglyceride	0.004 (0.002 to 0.006)	0.001	0.0016 (-0.0002 to 0.0033)	0.081
Particle size				
VLDL	-0.026 (-0.036 to -0.015)	<0.0001	-0.016 (-0.026 to -0.007)	0.0005
LDL	-0.017 (-0.049 to 0.015)	0.30	0.010 (-0.017 to 0.037)	0.47
HDL	-0.330 (-0.482 to -0.178)	<0.0001	-0.107 (-0.241 to 0.028)	0.12
Conventional lipid measures				
Total cholesterol	0.021 (-0.002 to 0.043)	0.068	0.015 (-0.004 to 0.034)	0.13
LDL cholesterol	0.015 (0.000 to 0.030)	0.049	0.007 (-0.005 to 0.020)	0.25
HDL cholesterol	-0.043 (-0.059 to -0.027)	<0.0001	-0.011 (-0.026 to 0.004)	0.14
Remnant cholesterol	0.027 (0.018 to 0.035)	<0.0001	0.009 (0.001 to 0.017)	0.024
Non-HDL cholesterol	0.030 (0.015 to 0.045)	0.0001	0.0128 (-0.0005 to 0.0260)	0.059
Triglyceride	0.027 (0.018 to 0.036)	<0.0001	0.009 (0.001 to 0.017)	0.027

For model 1, coefficients were adjusted for sex, age, current smoking, current alcohol assumption, blood glucose, while for model 2 coefficients were additionally adjusted for BMI. Coefficients were calculated for a doubling of the lipid concentration or 1 nm increment of the averaged lipoprotein particle size. Abbreviation: BMI, body mass index; HDL, high-density lipoprotein; LDL, low-density lipoprotein; VLDL, very-low-density lipoprotein; WHR, waist-to-hip ratio.

The association of WHR obesity with lipid parameters was similar, as shown in **Table 4**. In the multivariable-adjusted logistic regression models, WHR obesity was significantly associated with higher concentration of total VLDL particle, VLDL particle subclasses, triglycerides contents, and smaller VLDL particle size, independent of BMI and other covariables (P ≤ 0.021). The cholesterol and triglycerides contents, but not the concentration of HDL particles, were associated with WHR obesity (P ≤ 0.042). The associations between WHR and LDL particles concentrations, cholesterol and triglycerides contents, LDL particle size were null (P ≥ 0.28). Likewise, for these lipid parameters from the conventional lipid measurement, WHR obesity was associated with higher remnant cholesterol and triglycerides, but lower HDL cholesterol (P ≤ 0.013). The unadjusted associations of WHR and WHR obesity with lipid parameters are presented in supplementary **Tables S2**, **S3**.

**Table 4:** Association of WHR obesity with lipid parameters.

	WHR obesity vs. Normal WHR			
	Model 1: OR (95% CI)	P	Model 2: OR (95% CI)	P
Lipoprotein particle concentration				
VLDL particles				
Total	2.48 (1.89-3.26)	<0.0001	1.71 (1.28-2.29)	0.0003
Large	2.14 (1.59-2.88)	<0.0001	1.46 (1.06-2.01)	0.021
Medium	2.28 (1.77-2.93)	<0.0001	1.60 (1.22-2.10)	0.0007
Small	2.50 (1.91-3.28)	<0.0001	1.72 (1.29-2.31)	0.0002
Cholesterol	1.17 (1.09-1.25)	<0.0001	1.07 (0.99-1.15)	0.090
Triglyceride	2.99 (2.16-4.12)	<0.0001	1.94 (1.37-2.74)	0.0002
IDL particles				
Cholesterol	1.06 (1.00-1.14)	0.06	1.01 (0.93-1.08)	0.87
Triglyceride	1.17 (1.05-1.30)	0.00	1.09 (0.96-1.23)	0.18
LDL particles				
Total	0.99 (0.74-1.31)	0.93	0.93 (0.67-1.30)	0.68
Large	1.04 (0.79-1.36)	0.80	1.03 (0.74-1.42)	0.88
Medium	0.88 (0.66-1.16)	0.36	0.87 (0.62-1.21)	0.40
Small	1.02 (0.78-1.33)	0.90	0.94 (0.68-1.29)	0.71
Cholesterol	0.97 (0.76-1.24)	0.81	0.97 (0.73-1.30)	0.85
Triglyceride	1.00 (0.92-1.08)	0.89	0.97 (0.89-1.06)	0.49
HDL particles				
Total	0.79 (0.61-1.02)	0.07	0.88 (0.68-1.15)	0.35
Large	1.05 (0.89-1.25)	0.55	1.09 (0.90-1.31)	0.37
Medium	0.70 (0.55-0.91)	0.01	0.83 (0.65-1.07)	0.15
Small	0.83 (0.65-1.07)	0.15	0.91 (0.70-1.18)	0.47
Cholesterol	0.38 (0.24-0.58)	<0.0001	0.62 (0.39-0.98)	0.042
Triglyceride	1.14 (1.06-1.22)	0.00	1.09 (1.01-1.17)	0.038
Particle size				
VLDL	0.92 (0.88-0.96)	<0.0001	0.94 (0.90-0.98)	0.002
LDL	0.97 (0.86-1.09)	0.57	1.09 (0.93-1.28)	0.28
HDL	0.34 (0.19-0.60)	0.0002	0.61 (0.33-1.13)	0.12
Conventional lipid measures				
Total cholesterol	2.19 (1.02-4.70)	0.044	2.07 (0.86-4.96)	0.10
LDL cholesterol	1.90 (1.14-3.17)	0.014	1.67 (0.93-3.00)	0.089
HDL cholesterol	0.18 (0.10-0.33)	<0.0001	0.41 (0.21-0.79)	0.008
Remnant cholesterol	2.69 (1.91-3.78)	<0.0001	1.68 (1.16-2.43)	0.007
Non-HDL cholesterol	3.23 (1.86-5.59)	<0.0001	2.21 (1.18-4.14)	0.013
Triglyceride	2.70 (1.92-3.79)	<0.0001	1.67 (1.15-2.42)	0.007

For model 1, odds ratios (ORs) were adjusted for sex, age, current smoking, current alcohol assumption, blood glucose, while for model 2 ORs were additionally adjusted for BMI. ORs and 95% confidence intervals were calculated for a doubling of the lipid concentration or for a 0.1 nm increment of the averaged lipoprotein particle size. WHR obesity was defined as WHR ≥ 0.85 for women, 0.9 for men.

### Insulin sensitivity as a mediator in the association of obesity with lipoproteins

**Figure 1:**
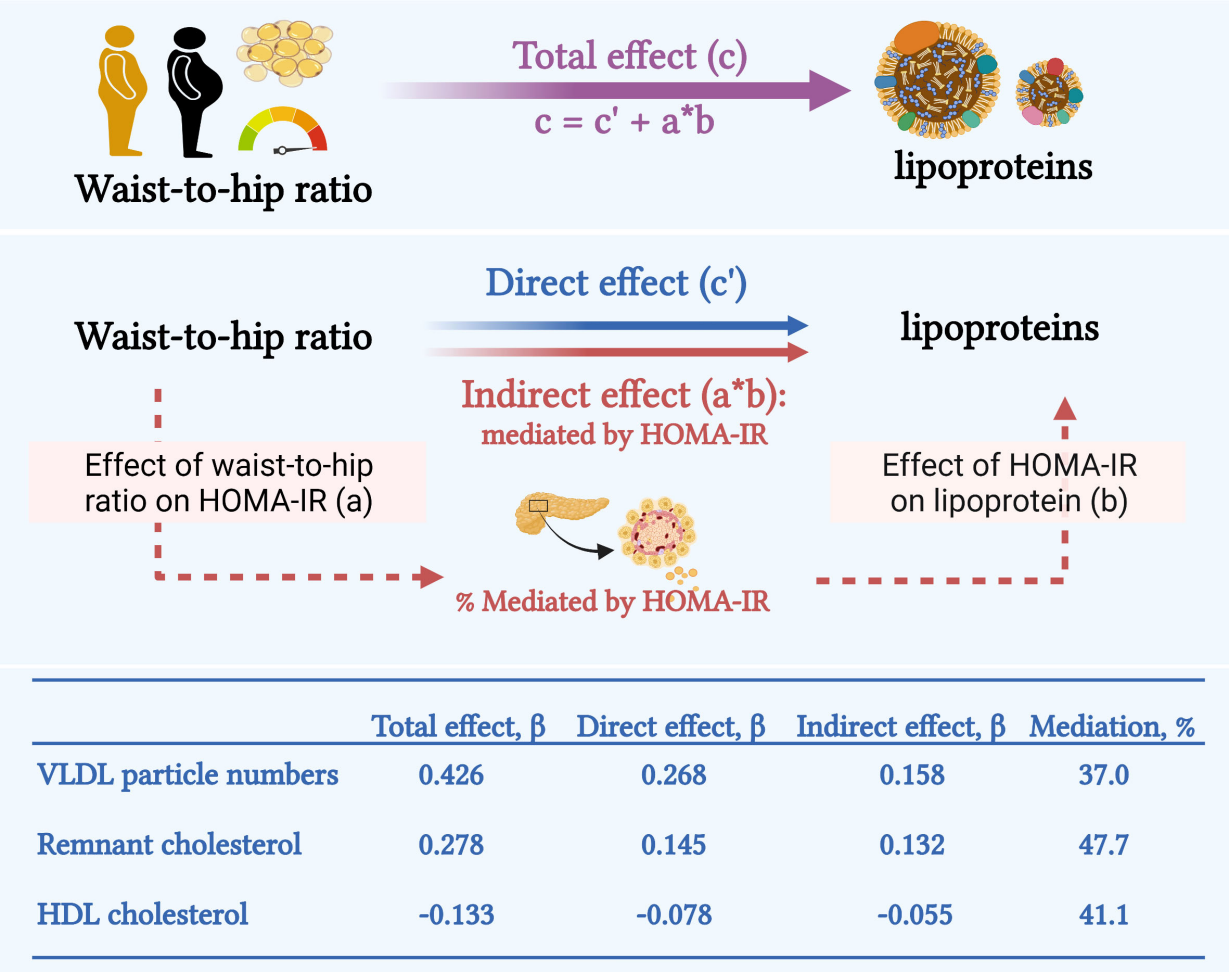
Insulin sensitivity mediated around 40-50% of the association of waist-to-hip ratio with VLDL particle concentration, remnant cholesterol, and HDL cholesterol. In the mediation model, HOMA-IR determined insulin sensitivity was considered a mediator between waist-to-hip ratio and VLDL particle concentration, remnant cholesterol, and HDL cholesterol. The effect of waist-to-hip ratio on HOMA-IR was defined as effect (a). The effect of HOMA-IR on a lipid parameter was defined as effect (b). The total effect of waist-to-hip ratio on a lipid parameter effect (c) comprised direct effect (c’) and indirect effect (a*b) mediated by HOMA-IR. These effects were assessed by β coefficients. The mediation percentages were the proportions explained by insulin sensitivity. All effects were adjusted for sex and age and P < 0.05. The figure was created with BioRender.com. HDL, high-density lipoprotein; HOMA-IR, homeostatic model assessment for insulin resistance; VLDL, very-low-density lipoprotein.

WHR was positively associated with HOMA-IR (β coefficient: 0.573, 95% CI: 0.443-0.702 for 0.1 increment in WHR), while HOMA-IR was positively associated with total VLDL particle concentration (β: 0.336, 95% CI: 0.270-0.403), remnant cholesterol (β: 0.264, 95% CI: 0.214-0.315), but was inversely associated with HDL cholesterol (β: -0.113, 95% CI: -0.142- -0.085) after adjustment of sex and age. Subsequently, the effect of WHR on these lipid parameters intermediated by HOMA-IR was assessed using mediation analysis, as illustrated in **Figure 1**. In mediation models, 37% of the effect of WHR on VLDL particle concentration was mediated by HOMA-IR (indirect effect: β=0.158 [95% CI: 0.104-0.211], P <0.0001). For remnant cholesterol and HDL cholesterol, the mediation percentage was 47.7% and 41.1% (P ≤ 0.0003), respectively.

### The relationship between 10-year cardiovascular risk score, WHR obesity, and lipid parameters

**Figure 2:**
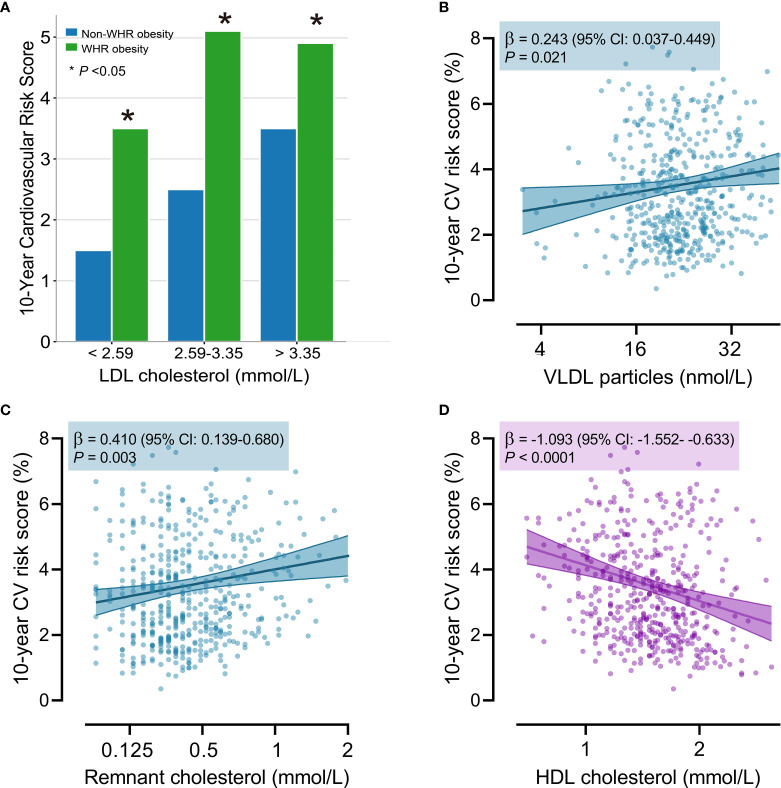
The relationship between 10-years cardiovascular risk score, WHR obesity, and lipid parameters. **(A)** The 10-years cardiovascular risk score across the LDL cholesterol categories and WHR obesity categories. The star (*) indicated a P-value < 0.05 between two groups in the same LDL cholesterol category. The linear association of cardiovascular risk with total VLDL particle concentration **(B)**, remnant cholesterol **(C)**, and HDL cholesterol **(D)**. The linear association was adjusted for sex and age. The solid line represents the regression line. The band with two solid lines indicates the 95% confidence limits of the regression line, and the transparent band refers to the 95% prediction limits of the regression model. β coefficients were calculated for a doubling of the lipid concentration. The 10-years cardiovascular risk score was estimated by the SCORE2 algorithm. HDL, high-density lipoprotein; LDL, low-density lipoprotein; VLDL, very-low-density lipoprotein; WHR, waist-to-hip ratio.

**Figure 2** shows the 10-year cardiovascular risk score across the LDL cholesterol categories and WHR obesity categories. Individuals with higher LDL cholesterol were at an increased cardiovascular risk, independent of WHR obesity. However, regardless of the LDL cholesterol categories, individuals with WHR obesity had a consistently higher cardiovascular risk score compared with those without WHR obesity (P ≤0.028). This might indicate a residual cardiovascular risk for individuals with WHR obesity. Besides, WHR-associated lipid parameters showed a significant association with the estimated cardiovascular risk. As shown by **Figure 2**, VLDL particle concentrations and remnant cholesterol, positively associated with WHR, were significantly associated with an increased cardiovascular risk (β=0.261, 95% CI: 0.047-0.475 and β=0.434, 95% CI: 0.156-0.713, P≤ 0.002), whereas HDL particle concentration, inversely associated with WHR, presented a negative association with cardiovascular risk score (β=-1.330, 95% CI: -1.836- -0.825, P <0.0001).

## Discussion

The main findings of the current study can be summarized as 1) WHR-defined obesity was far more prevalent than BMI-defined obesity, and individuals with WHR obesity had higher metabolic complications compared to those with normal WHR; 2) the prominent lipid alterations associated with WHR included increased VLDL particle concentration and remnant cholesterol, and decreased HDL particle concentration, rather than LDL particle concentration or LDL cholesterol; 3) insulin sensitivity mediated roughly 40-50% of the association between WHR and the major altered lipid parameters; 4) individuals with WHR obesity were at higher cardiovascular risk and the WHR obesity-associated lipoprotein alteration was associated an increased cardiovascular risk.

The lipoprotein profile of WHR obesity is consistent with previous findings that abdominal obesity is associated with atherogenic lipid alteration (8). Visceral abdominal fat determined by computerized tomography scan was associated with higher VLDL particle concentration and LDL particle concentration, and smaller LDL particles and HDL particles (8). Our study further suggested the similarity of the association of lipoproteins with upper-body fat distribution and overall fat deposition. Even if BMI is prevailingly used to define obesity, BMI remarkably underestimates the prevalence of abdominal obesity. Moreover, a previous study suggested that WHR provided the highest sensitivity (94.1% for males and 86.7% for females) for the detection of metabolic syndrome in 1104 participants, outperforming both BMI and waist circumference (30). Therefore, WHR is a promising alternative to identify abdominal obesity and metabolic syndrome carriers, delivered by a feasible, simple anthropometric measurement. In line with the solid evidence that WHR is independently associated with mortality and cardiovascular risk (4–6, 31, 32), our findings also supported that WHR obesity seemed to capture the residual cardiovascular risk on top of LDL cholesterol. We found that the estimated cardiovascular risk was consistently higher in individuals with WHR obesity, even with optimal LDL cholesterol levels. The application of WHR may pave the way for early intervention of abdominal obesity and properly assess residual cardiovascular risk for individuals with central body fat distribution.

The present finding also confirmed that VLDL particle concentration was more strongly related to fat distribution as compared to LDL particle concentration. The underlying clinical relevance is that LDL cholesterol might underrate the lipid abnormality in people with upper-body obesity, and VLDL-derived lipid parameters would be more informative. For instance, we found that remnant cholesterol, an estimate for the cholesterol of VLDL particles and VLDL particle remnants, was consistently associated with upper-body obesity. High remnant cholesterol has been demonstrated to be associated with an increased cardiovascular risk (33, 34). In this study, we also observed the positive association between remnant cholesterol and the estimated cardiovascular risk score. High remnant cholesterol may partly explain the residual cardiovascular risk in people with abdominal obesity, even when LDL cholesterol levels are optimal. Noteworthy, remnant cholesterol could be estimated by the existing conventional lipid measures without extra cost. Another lipid parameter derived from VLDL particles is triglycerides. Although elevated triglycerides are the hallmark of the lipid abnormality of obesity, the association of triglycerides per se with cardiovascular risk has been debated for decades (35–37). The altered lipid metabolism characterization in people with obesity might be responsible for the insignificant association between LDL-cholesterol and abdominal obesity. Another possible explanation may relate to the influence of genetic factors on LDL-cholesterol. Among genetic lipid disorders, LDL-cholesterol is more commonly affected (38, 39). Large-scale genome-wide studies integrated the genetic variants associated with LDL-cholesterol with polygenic risk scores responsible for 11-21% of the variance of LDL-cholesterol (40, 41). The correlation between the polygenic risk score and HDL was 0.11 (41).

Apart from VLDL particles, smaller LDL particles and HDL particles are prominent factors in relation to obesity. With the persistent status of high triglyceride-enriched VLDL particle concentration in people with obesity, cholesteryl ester transfer protein (CETP) promotes the exchange of cholesteryl esters from LDL particles and HDL particles for triglycerides from VLDL particles (42). The growing triglycerides contents in LDL and HDL particles tend to be hydrolyzed by hepatic lipase, which generates small, dense LDL and HDL particles. Smaller LDL particles are prone to deposit within arteries; thus, they are atherogenic and associated with the risk of cardiovascular disease (42–45). By contrast, smaller HDL particles contain fewer cholesterol contents, and the smaller particle size is susceptible to degradation by lipases, leading to decreased HDL cholesterol (46).

Our study found that insulin sensitivity is an essential mediator in the association between fat distribution and dyslipidemia, which is in the agreement with previous findings (47, 48). On the one hand, abdominal obesity has been strongly associated with insulin sensitivity and the mechanistic links are considered multifactorial (49). The hypertrophic adipocytes and adipose tissues induce pre-inflammatory cytokines, such as tumor necrosis factor α (TNF-α), that prevent insulin signaling and lead to insulin resistance (46). The impaired free fatty acids storage of enlarged adipose tissues increases free fatty acids in circulation and leads to ectopic fat deposition in the pancreas that dysregulates β-cells and contributes to insulin resistance as well (50, 51). On the other hand, insulin resistance plays a pivotal role in the pathogenesis of obesity-induced lipid disorders, especially in the overproduction of VLDL particles and the reduction of HDL particles (47, 48). The role of insulin resistance can be found in diverse pathways. For instance, an interesting study recently reported that the link between HOMA-IR and the level of PCSK9 is evident in people with obesity (52). Moreover, the effect of depression on HOMA-IR was partially mediated by PCSK9 level, providing a potential treatment strategy for the improvement of insulin sensitivity (52). Based on the existed evidence, our findings emphasized the significance of insulin sensitivity in the development of lipid abnormality in people with upper-body obesity. Given the mediation percentage (40-50%) of insulin sensitivity, the lifestyle modifications, such as weight loss and carbohydrate-restricted diets, might effectively intervene against dyslipidemia in individuals with obesity on multiple levels, as it has been associated with the improvement in both obesity and insulin sensitivity (53, 54).

Along with adopting healthy lifestyles, restrictive dietary strategies have been reported to influence cardiovascular risk. Long-term fasting (14 days, daily calorie intake: 200–250 kcal) can limit the generation of chylomicrons, stimulate the mobilization of fatty acids from the adipose tissue, and subsequently relate to decreased triglyceride-enriched VLDL particles and LDL cholesterol, as well as higher large HDL particles (55). The lipoprotein profile improved by fasting is less atherogenic and associated with lower cardiovascular risk (55). Pharmacological therapy is another option for lipid-lowering therapy in people with obesity. Statins are the first-line lipid-lowering option to reduce LDL cholesterol and cardiovascular risk (36). However, the efficiency of statins on triglycerides is marginal (36). Given the association of fat distribution with lipoprotein constitutions, specific treatments targeting the elevated VLDL particle concentration and remnant cholesterol hold the potentiality to reduce the remaining cardiovascular risks in individuals with abdominal obesity. It was supported by a large clinical trial of 9423 participants. In this clinical trial, the reduction of VLDL particles with statin was found to associate with reduced risk of atherosclerotic cardiovascular disease, independent of the existence of low LDL cholesterol (56). The proprotein convertase subtilisin/kexin type 9 (PCSK9) inhibitors provide an alternative approach to lower LDL cholesterol. However, its effect on VLDL particle concentration or remnant cholesterol remains inconclusive (36). A large randomized, controlled trial (PROSPER) recently compared the effect of pravastatin (40 mg/d) and a loss-of-mutation in PCSK9 gene on NMR spectrometry-measured lipoprotein profile, and it demonstrated that PCSK9 inhibition has a weaker effect on VLDL particle concentration (57). In parallel, alirocumab, a monoclonal antibody of PCSK9 was shown to have no impact on the metabolism of VLDL particles (58). These findings contradicted with other reports on monoclonal antibodies of PCSK9 (59, 60). A real-world study in 350 patients receiving PCSK9 antibodies (alirocumab or evolocumab) suggested that PCSK9 inhibitors reduce small VLDL particle concentration (60). Moreover, targeting angiopoietin-like protein 3 (ANGPTL3) shows the potentiality to effectively reduce VLDL particle concentration (reduction of 27.9 to 60.0%), remnant cholesterol (reduction of 38%), and cardiovascular risk (61–63).

A strength of our study is the inclusion of well-characterized participants from the general population since the observed early association might be involved in the development and prevention of cardiometabolic complications. Other major strengths included the incorporation of NMR spectrometry-measured lipoproteins and conventional lipid measures, the application of multivariate analysis with the adjustment of potential confounders, and mediation analysis to identify potential mechanisms for the association of obesity and dyslipidemia. This study also has several limitations. As a cross-sectional study, the observed association between waist-to-hip ratio and atherogenic lipid profile cannot prove causality. Furthermore, given the atherogenic property of lipoprotein (a), accumulating evidence has revealed the causality between long-term exposure to higher lipoprotein (a) and increased risk of cardiovascular events (36). However, the lipoprotein profile of our study did not measure lipoprotein (a) because it was not included in the measurements protocol when the participants were recruited. Besides, cardiovascular risk was not calculated by cardiovascular events in the follow-up but was estimated by the SCORE2 algorithm which includes clinical and biochemical risk factors. SCORE2 is not validated for patients with diabetes or with already known cardiovascular disease, where it underestimates risk. However, the prevalence of diabetes and previous cardiovascular diseases in the studied population was 1.6% and 5.8%, respectively. The findings from sensitivity analyses excluding these participants were confirmative.

## Conclusions

WHR obesity, more prevalent than BMI-determined obesity, was independently associated with an unfavorable lipoprotein profile. Insulin sensitivity was a pivotal mediator that links upper-body fat deposition to lipid disorders. VLDL particle concentration and remnant cholesterol are more strongly associated with obesity than LDL cholesterol. Lowering VLDL particle concentration and remnant cholesterol might further reduce the residual cardiovascular risk for individuals with obesity.

## Data availability statement

The original contributions presented in the study are included in the article/**Supplementary Material**.

## Ethics statement

The studies involving human participants were reviewed and approved by The University of Leuven Ethics Committee. Written informed consent to participate in this study was provided by the participants and the participants' legal guardian/next of kin.

## Author contributions

DW and Z-YZ conceptualized and designed the study. VM, DM, JR, and Z-YZ contributed to data acquisition. DW and Z-YZ performed analysis. All authors interpreted the data. DW initially drafted the manuscript. DW, LV, TV, and Z-YZ critically revised the manuscript. All authors reviewed and approved the final manuscript.

## Funding

The European Research Area Net for Cardiovascular Diseases (JTC2017-046-PROACT), the Ministerio de Ciencia e Innovación of Spain (PID2019-108973RB-C22 and PCIN2017-117), the Generalitat Valenciana of Spain (GV/2020/048), and GUTMOM (INTIMIC-085) from the EU Joint Programming Initiative Healthy Diet Healthy Life (HDHL). The Internal Funds KU Leuven (STG-18-00379) currently supports the Studies Coordinating Centre in Leuven.

## Acknowledgments

The authors acknowledge the clerical contribution of Renilde Wolfs.

## Conflict of interest

The authors declare that the research was conducted in the absence of any commercial or financial relationships that could be construed as a potential conflict of interest.

## Publisher’s note

All claims expressed in this article are solely those of the authors and do not necessarily represent those of their affiliated organizations, or those of the publisher, the editors and the reviewers. Any product that may be evaluated in this article, or claim that may be made by its manufacturer, is not guaranteed or endorsed by the publisher.

## References

[1] Collaboration NCDRF. Worldwide trends in body-mass index, underweight, overweight, and obesity from 1975 to 2016: A pooled analysis of 2416 population-based measurement studies in 128.9 million children, adolescents, and adults. Lancet (2017) 390(10113):2627–42. doi: 10.1016/S0140-6736(17)32129-3 PMC5735219 29029897

[2] Bluher M. Obesity: Global epidemiology and pathogenesis. Nat Rev Endocrinol (2019) 15(5):288–98. doi: 10.1038/s41574-019-0176-8 30814686

[3] Tsujimoto T Kajio H. Abdominal obesity is associated with an increased risk of all-cause mortality in patients with hfpef. J Am Coll Cardiol (2017) 70(22):2739–49. doi: 10.1016/j.jacc.2017.09.1111 29191321

[4] de Koning L Merchant AT Pogue J Anand SS. Waist circumference and waist-to-Hip ratio as predictors of cardiovascular events: Meta-regression analysis of prospective studies. Eur Heart J (2007) 28(7):850–6. doi: 10.1093/eurheartj/ehm026 17403720

[5] Lakka HM Lakka TA Tuomilehto J Salonen JT. Abdominal obesity is associated with increased risk of acute coronary events in men. Eur Heart J (2002) 23(9):706–13. doi: 10.1053/euhj.2001.2889 11977996

[6] O'Donnell MJ Chin SL Rangarajan S Xavier D Liu L Zhang H. Global and regional effects of potentially modifiable risk factors associated with acute stroke in 32 countries (Interstroke): A case-control study. Lancet (2016) 388(10046):761–75. doi: 10.1016/S0140-6736(16)30506-2 27431356

[7] Racette SB Evans EM Weiss EP Hagberg JM Holloszy JO. Abdominal adiposity is a stronger predictor of insulin resistance than fitness among 50-95 year olds. Diabetes Care (2006) 29(3):673–8. doi: 10.2337/diacare.29.03.06.dc05-1605 PMC4376197 16505525

[8] Sam S Haffner S Davidson MH D'Agostino RB C.OMMAS.R.X.X.X. Feinstein S Kondos G. Relationship of abdominal visceral and subcutaneous adipose tissue with lipoprotein particle number and size in type 2 diabetes. Diabetes (2008) 57(8):2022–7. doi: 10.2337/db08-0157 PMC2494673 18469202

[9] Magkos F Mohammed BS Mittendorfer B. Effect of obesity on the plasma lipoprotein subclass profile in normoglycemic and normolipidemic men and women. Int J Obes (Lond) (2008) 32(11):1655–64. doi: 10.1038/ijo.2008.164 PMC2584161 18779822

[10] Guardiola M Sola R Vallve JC Girona J Godas G Heras M. Body mass index correlates with atherogenic lipoprotein profile even in nonobese, normoglycemic, and normolipidemic healthy men. J Clin Lipidol (2015) 9(6):824–31.e1. doi: 10.1016/j.jacl.2015.08.001 26687704

[11] Zaid M Miura K Okayama A Nakagawa H Sakata K Saitoh S. Associations of high-density lipoprotein particle and high-density lipoprotein cholesterol with alcohol intake, smoking, and body mass index- the interlipid study. Circ J (2018) 82(10):2557–65. doi: 10.1253/circj.CJ-18-0341 PMC6688485 30135319

[12] Prospective Studies Collaboration Whitlock G Lewington S Sherliker P Clarke R Emberson J. Body-mass index and cause-specific mortality in 900 000 adults: Collaborative analyses of 57 prospective studies. Lancet (2009) 373(9669):1083–96. doi: 10.1016/S0140-6736(09)60318-4 PMC2662372 19299006

[13] Emerging Risk Factors Collaboration Wormser D Kaptoge S Di Angelantonio E AM W Pennells L. Separate and combined associations of body-mass index and abdominal adiposity with cardiovascular disease: Collaborative analysis of 58 prospective studies. Lancet (2011) 377(9771):1085–95. doi: 10.1016/S0140-6736(11)60105-0 PMC3145074 21397319

[14] Yusuf S Hawken S Ounpuu S Bautista L Franzosi MG Commerford P. Obesity and the risk of myocardial infarction in 27,000 participants from 52 countries: A case-control study. Lancet (2005) 366(9497):1640–9. doi: 10.1016/S0140-6736(05)67663-5 16271645

[15] Hertelyova Z Salaj R Chmelarova A Dombrovsky P Dvorakova MC Kruzliak P. The association between lipid parameters and obesity in university students. J Endocrinol Invest (2016) 39(7):769–78. doi: 10.1007/s40618-015-0240-8 25601518

[16] Gourgari E Lodish M Shamburek R Keil M Wesley R Walter M. Lipoprotein particles in adolescents and young women with pcos provide insights into their cardiovascular risk. J Clin Endocrinol Metab (2015) 100(11):4291–8. doi: 10.1210/jc.2015-2566 PMC4702461 26371381

[17] Hodge AM Jenkins AJ English DR O'Dea K Giles GG. Nmr-determined lipoprotein subclass profile is associated with dietary composition and body size. Nutr Metab Cardiovasc Dis (2011) 21(8):603–9. doi: 10.1016/j.numecd.2009.12.003 21084180

[18] James RW Brulhart-Meynet MC Lehmann T Golay A. Lipoprotein distribution and composition in obesity: Their association with central adiposity. Int J Obes Relat Metab Disord (1997) 21(12):1115–20. doi: 10.1038/sj.ijo.0800524 9426378

[19] Garvey WT Kwon S Zheng D Shaughnessy S Wallace P Hutto A. Effects of insulin resistance and type 2 diabetes on lipoprotein subclass particle size and concentration determined by nuclear magnetic resonance. Diabetes (2003) 52(2):453–62. doi: 10.2337/diabetes.52.2.453 12540621

[20] Goff DC Jr. D'Agostino RB Jr. Haffner SM Otvos JD. Insulin resistance and adiposity influence lipoprotein size and subclass concentrations. results from the insulin resistance atherosclerosis study. Metabolism (2005) 54(2):264–70. doi: 10.1016/j.metabol.2004.09.002 15690322

[21] Zhang ZY Thijs L Petit T Gu YM Jacobs L Yang WY. Urinary proteome and systolic blood pressure as predictors of 5-year cardiovascular and cardiac outcomes in a general population. Hypertension (2015) 66(1):52–60. doi: 10.1161/HYPERTENSIONAHA.115.05296 26063667

[22] World Health Organization. Waist circumference and waist-hip ratio: Report of a who expert consultation. Geneva: World Health Organization (2011) p. 8–11. Available at: https://apps.who.int/iris/handle/10665/44583.

[23] Mallol R Amigo N Rodriguez MA Heras M Vinaixa M Plana N. Liposcale: A novel advanced lipoprotein test based on 2d diffusion-ordered 1h nmr spectroscopy. J Lipid Res (2015) 56(3):737–46. doi: 10.1194/jlr.D050120 PMC4340320 25568061

[24] Mallol R Rodriguez MA Heras M Vinaixa M Plana N Masana L. Particle size measurement of lipoprotein fractions using diffusion-ordered nmr spectroscopy. Anal Bioanal Chem (2012) 402(7):2407–15. doi: 10.1007/s00216-011-5705-9 22293969

[25] National Cholesterol Education Program (NCEP) Expert Panel on Detection, Evaluation, and Treatment of High Blood Cholesterol in Adults (Adult Treatment Panel III). Third report of the national cholesterol education program (Ncep) expert panel on detection, evaluation, and treatment of high blood cholesterol in adults (Adult treatment panel iii) final report. Circulation (2002) 106(25):3143–421. doi: 10.1161/circ.106.25.3143 12485966

[26] Stevens LA Claybon MA Schmid CH Chen J Horio M Imai E. Evaluation of the chronic kidney disease epidemiology collaboration equation for estimating the glomerular filtration rate in multiple ethnicities. Kidney Int (2011) 79(5):555–62. doi: 10.1038/ki.2010.462 PMC4220293 21107446

[27] SCORE2-OP working group and ESC Cardiovascular risk collaboration. Score2 risk prediction algorithms: New models to estimate 10-year risk of cardiovascular disease in Europe. Eur Heart J (2021) 42(25):2439–54. doi: 10.1093/eurheartj/ehab309 PMC8248998 34120177

[28] Phillips CM Perry IJ. Lipoprotein particle subclass profiles among metabolically healthy and unhealthy obese and non-obese adults: Does size matter? Atherosclerosis (2015) 242(2):399–406. doi: 10.1016/j.atherosclerosis.2015.07.040 26277632

[29] Otvos JD Collins D Freedman DS Shalaurova I Schaefer EJ McNamara JR. Low-density lipoprotein and high-density lipoprotein particle subclasses predict coronary events and are favorably changed by gemfibrozil therapy in the veterans affairs high-density lipoprotein intervention trial. Circulation (2006) 113(12):1556–63. doi: 10.1161/CIRCULATIONAHA.105.565135 16534013

[30] Pavanello C Zanaboni AM Gaito S Botta M Mombelli G Sirtori CR. Influence of body variables in the development of metabolic syndrome-a long term follow-up study. PloS One (2018) 13(2):e0192751. doi: 10.1371/journal.pone.0192751 29432480 PMC5809080

[31] Jayedi A Soltani S Zargar MS Khan TA Shab-Bidar S. Central fatness and risk of all cause mortality: Systematic review and dose-response meta-analysis of 72 prospective cohort studies. Bmj (2020) 370:m3324. doi: 10.1136/bmj.m3324 32967840 PMC7509947

[32] Postorino M Marino C Tripepi G Zoccali C Group CW. Abdominal obesity and all-cause and cardiovascular mortality in end-stage renal disease. J Am Coll Cardiol (2009) 53(15):1265–72. doi: 10.1016/j.jacc.2008.12.040 19358939

[33] Castaner O Pinto X Subirana I Amor AJ Ros E Hernaez A. Remnant cholesterol, not ldl cholesterol, is associated with incident cardiovascular disease. J Am Coll Cardiol (2020) 76(23):2712–24. doi: 10.1016/j.jacc.2020.10.008 33272365

[34] Varbo A Nordestgaard BG. Directly measured vs. calculated remnant cholesterol identifies additional overlooked individuals in the general population at higher risk of myocardial infarction. Eur Heart J (2021) 42(47):4833–43. doi: 10.1093/eurheartj/ehab293 34023898

[35] Nordestgaard BG. Triglyceride-rich lipoproteins and atherosclerotic cardiovascular disease: New insights from epidemiology, genetics, and biology. Circ Res (2016) 118(4):547–63. doi: 10.1161/CIRCRESAHA.115.306249 26892957

[36] Mach F Baigent C Catapano AL Koskinas KC Casula M Badimon L. 2019 Esc/Eas guidelines for the management of dyslipidaemias: Lipid modification to reduce cardiovascular risk. Eur Heart J (2020) 41(1):111–88. doi: 10.1093/eurheartj/ehz455 31504418

[37] Reiner Z. Hypertriglyceridaemia and risk of coronary artery disease. Nat Rev Cardiol (2017) 14(7):401–11. doi: 10.1038/nrcardio.2017.31 28300080

[38] Kronenberg F Mora S Stroes ESG Ference BA Arsenault BJ Berglund L. Lipoprotein(a) in atherosclerotic cardiovascular disease and aortic stenosis: A European atherosclerosis society consensus statement. Eur Heart J (2022) 43(39):3925–46. doi: 10.1093/eurheartj/ehac361 PMC9639807 36036785

[39] Reyes-Soffer G Ginsberg HN Berglund L Duell PB Heffron SP Kamstrup PR. Lipoprotein(a): A genetically determined, causal, and prevalent risk factor for atherosclerotic cardiovascular disease: A scientific statement from the American heart association. Arterioscler Thromb Vasc Biol (2022) 42(1):e48–60. doi: 10.1161/ATV.0000000000000147 PMC9989949 34647487

[40] Talmud PJ Shah S Whittall R Futema M Howard P Cooper JA. Use of low-density lipoprotein cholesterol gene score to distinguish patients with polygenic and monogenic familial hypercholesterolaemia: A case-control study. Lancet (2013) 381(9874):1293–301. doi: 10.1016/S0140-6736(12)62127-8 23433573

[41] Wu H Forgetta V Zhou S Bhatnagar SR Pare G Richards JB. Polygenic risk score for low-density lipoprotein cholesterol is associated with risk of ischemic heart disease and enriches for individuals with familial hypercholesterolemia. Circ Genom Precis Med (2021) 14(1):e003106. doi: 10.1161/CIRCGEN.120.003106 33440130

[42] Tchernof A Despres JP. Pathophysiology of human visceral obesity: An update. Physiol Rev (2013) 93(1):359–404. doi: 10.1152/physrev.00033.2011 23303913

[43] Mora S Otvos JD Rifai N Rosenson RS Buring JE Ridker PM. Lipoprotein particle profiles by nuclear magnetic resonance compared with standard lipids and apolipoproteins in predicting incident cardiovascular disease in women. Circulation (2009) 119(7):931–9. doi: 10.1161/CIRCULATIONAHA.108.816181 PMC2663974 19204302

[44] Kuller L Arnold A Tracy R Otvos J Burke G Psaty B. Nuclear magnetic resonance spectroscopy of lipoproteins and risk of coronary heart disease in the cardiovascular health study. Arterioscler Thromb Vasc Biol (2002) 22(7):1175–80. doi: 10.1161/01.atv.0000022015.97341.3a 12117734

[45] El Harchaoui K van der Steeg WA Stroes ES Kuivenhoven JA Otvos JD Wareham NJ. Value of low-density lipoprotein particle number and size as predictors of coronary artery disease in apparently healthy men and women: The epic-Norfolk prospective population study. J Am Coll Cardiol (2007) 49(5):547–53. doi: 10.1016/j.jacc.2006.09.043 17276177

[46] Bays HE Toth PP Kris-Etherton PM Abate N Aronne LJ Brown WV. Obesity, adiposity, and dyslipidemia: A consensus statement from the national lipid association. J Clin Lipidol (2013) 7(4):304–83. doi: 10.1016/j.jacl.2013.04.001 23890517

[47] Ormazabal V Nair S Elfeky O Aguayo C Salomon C Zuniga FA. Association between insulin resistance and the development of cardiovascular disease. Cardiovasc Diabetol (2018) 17(1):122. doi: 10.1186/s12933-018-0762-4 30170598 PMC6119242

[48] Sparks JD Sparks CE Adeli K. Selective hepatic insulin resistance, vldl overproduction, and hypertriglyceridemia. Arterioscler Thromb Vasc Biol (2012) 32(9):2104–12. doi: 10.1161/ATVBAHA.111.241463 22796579

[49] Neeland IJ Poirier P Despres JP. Cardiovascular and metabolic heterogeneity of obesity: Clinical challenges and implications for management. Circulation (2018) 137(13):1391–406. doi: 10.1161/CIRCULATIONAHA.117.029617 PMC5875734 29581366

[50] Wong VW Wong GL Yeung DK Abrigo JM Kong AP Chan RS. Fatty pancreas, insulin resistance, and beta-cell function: A population study using fat-water magnetic resonance imaging. Am J Gastroenterol (2014) 109(4):589–97. doi: 10.1038/ajg.2014.1 24492753

[51] Wu WC Wang CY. Association between non-alcoholic fatty pancreatic disease (Nafpd) and the metabolic syndrome: Case-control retrospective study. Cardiovasc Diabetol (2013) 12:77. doi: 10.1186/1475-2840-12-77 23688357 PMC3682938

[52] Macchi C Favero C Ceresa A Vigna L Conti DM Pesatori AC. Depression and cardiovascular risk-association among beck depression inventory, Pcsk9 levels and insulin resistance. Cardiovasc Diabetol (2020) 19(1):187. doi: 10.1186/s12933-020-01158-6 33143700 PMC7641831

[53] Clamp LD Hume DJ Lambert EV Kroff J. Enhanced insulin sensitivity in successful, long-term weight loss maintainers compared with matched controls with no weight loss history. Nutr Diabetes (2017) 7(6):e282. doi: 10.1038/nutd.2017.31 28628125 PMC5519190

[54] Thomsen MN Skytte MJ Samkani A Carl MH Weber P Astrup A. Dietary carbohydrate restriction augments weight loss-induced improvements in glycaemic control and liver fat in individuals with type 2 diabetes: A randomised controlled trial. Diabetologia (2022) 65(3):506–17. doi: 10.1007/s00125-021-05628-8 PMC8739348 34993571

[55] Grundler F Plonne D Mesnage R Muller D Sirtori CR Ruscica M. Long-term fasting improves lipoprotein-associated atherogenic risk in humans. Eur J Nutr (2021) 60(7):4031–44. doi: 10.1007/s00394-021-02578-0 PMC8437871 33963431

[56] Lawler PR Akinkuolie AO Harada P Glynn RJ Chasman DI Ridker PM. Residual risk of atherosclerotic cardiovascular events in relation to reductions in very-Low-Density lipoproteins. J Am Heart Assoc (2017) 6(12):e007402. doi: 10.1161/JAHA.117.007402 29223956 PMC5779048

[57] Sliz E Kettunen J Holmes MV Williams CO Boachie C Wang Q. Metabolomic consequences of genetic inhibition of Pcsk9 compared with statin treatment. Circulation (2018) 138(22):2499–512. doi: 10.1161/CIRCULATIONAHA.118.034942 PMC6254781 30524137

[58] Reyes-Soffer G Pavlyha M Ngai C Thomas T Holleran S Ramakrishnan R. Effects of Pcsk9 inhibition with alirocumab on lipoprotein metabolism in healthy humans. Circulation (2017) 135(4):352–62. doi: 10.1161/CIRCULATIONAHA.116.025253 PMC5262523 27986651

[59] Toth PP Sattar N Blom DJ Martin SS Jones SR Monsalvo ML. Effect of evolocumab on lipoprotein particles. Am J Cardiol (2018) 121(3):308–14. doi: 10.1016/j.amjcard.2017.10.028 29221604

[60] Hollstein T Vogt A Grenkowitz T Stojakovic T Marz W Laufs U. Treatment with Pcsk9 inhibitors reduces atherogenic vldl remnants in a real-world study. Vascul Pharmacol (2019) 116:8–15. doi: 10.1016/j.vph.2019.03.002 30910670

[61] Gaudet D Karwatowska-Prokopczuk E Baum SJ Hurh E Kingsbury J Bartlett VJ. Vupanorsen, an n-acetyl galactosamine-conjugated antisense drug to Angptl3 mrna, lowers triglycerides and atherogenic lipoproteins in patients with diabetes, hepatic steatosis, and hypertriglyceridaemia. Eur Heart J (2020) 41(40):3936–45. doi: 10.1093/eurheartj/ehaa689 PMC7750927 32860031

[62] Dewey FE Gusarova V Dunbar RL O'Dushlaine C Schurmann C Gottesman O. Genetic and pharmacologic inactivation of Angptl3 and cardiovascular disease. N Engl J Med (2017) 377(3):211–21. doi: 10.1056/NEJMoa1612790 PMC5800308 28538136

[63] Graham MJ Lee RG Brandt TA Tai LJ Fu W Peralta R. Cardiovascular and metabolic effects of Angptl3 antisense oligonucleotides. N Engl J Med (2017) 377(3):222–32. doi: 10.1056/NEJMoa1701329 28538111

