# Supplementary material for: Lipoprotein profiles of fat distribution and its association with insulin sensitivity
Source: Front Endocrinol (Lausanne). 2022 Oct 25;13:978745. doi: 10.3389/fendo.2022.978745 (PMC9640977; doi:10.3389/fendo.2022.978745)
Supplement: Supplementary file 1 [file DataSheet_1.docx]

Supplementary Material

# Supplementary Tables

## Table S1. Correlations of Lipoprotein Parameters with Conventional Lipid Measures

|  | **Total cholesterol** | **LDL**  **cholesterol** | **HDL**  **cholesterol** | **Remnant**  **cholesterol** | **Non-HDL LDL**  **cholesterol** | **Triglycerides** |
| --- | --- | --- | --- | --- | --- | --- |
| VLDL particles |  |  |  |  |  |  |
| Total | 0.268 | 0.280 | -0.504 | 0.914 | 0.492 | 0.913 |
| Large | 0.275 | 0.299 | -0.490 | 0.857 | 0.490 | 0.857 |
| Medium | 0.261 | 0.275 | -0.510 | 0.908 | 0.487 | 0.907 |
| Small | 0.269 | 0.280 | -0.500 | 0.912 | 0.491 | 0.911 |
| Cholesterol | 0.286 | 0.309 | -0.511 | 0.882 | 0.510 | 0.881 |
| TG | 0.261 | 0.269 | -0.498 | 0.909 | 0.481 | 0.909 |
| IDL particles |  |  |  |  |  |  |
| Cholesterol | 0.550 | 0.452 | -0.043* | 0.634 | 0.566 | 0.636 |
| TG | 0.425 | 0.304 | -0.030* | 0.606 | 0.427 | 0.610 |
| LDL particles |  |  |  |  |  |  |
| Total | 0.776 | 0.723 | 0.219 | 0.285 | 0.677 | 0.286 |
| Large | 0.738 | 0.682 | 0.278 | 0.206 | 0.618 | 0.208 |
| Medium | 0.741 | 0.718 | 0.202 | 0.230 | 0.651 | 0.233 |
| Small | 0.781 | 0.711 | 0.209 | 0.331 | 0.686 | 0.332 |
| Cholesterol | 0.772 | 0.746 | 0.235 | 0.222 | 0.670 | 0.224 |
| TG | 0.595 | 0.484 | 0.186 | 0.307 | 0.503 | 0.310 |
| HDL particles |  |  |  |  |  |  |
| Total | 0.103 | -0.188 | 0.608 | -0.142 | -0.174 | -0.141 |
| Large | 0.183 | 0.128 | 0.156 | 0.105 | 0.118 | 0.107 |
| Medium | -0.007* | -0.286 | 0.699 | -0.332 | -0.316 | -0.332 |
| Small | 0.152 | -0.134 | 0.542 | -0.041* | -0.099 | -0.040* |
| Cholesterol | 0.031* | -0.219 | 0.747 | -0.403 | -0.295 | -0.403 |
| TG | 0.101 | -0.003* | -0.18 | 0.564 | 0.166 | 0.565 |
| Particle size |  |  |  |  |  |  |
| VLDL particles | -0.024* | 0.028* | 0.030* | -0.192 | -0.043* | -0.192 |
| LDL particles | -0.063* | 0.015* | 0.164 | -0.411 | -0.125 | -0.409 |
| HDL particles | -0.239 | -0.302 | 0.504 | -0.672 | -0.454 | -0.670 |

The correlations were significant (P <0.05) unless specifically indicated with *. Abbreviation: HDL, high-density lipoprotein; IDL, intermediate-density lipoprotein; LDL, low-density lipoprotein; TG, triglycerides; TP: total particle; VLDL, very low-density lipoprotein; WC, waist circumference; WHR, waist-to-hip ratio.

## Table S2. Unadjusted Associations of WHR with Lipid Parameters

|  | **WHR** | |
| --- | --- | --- |
|  | **Unadjusted coefficient** | **P** |
| VLDL particles |  |  |
| Total | 0.038 (0.030 to 0.045) | <0.0001 |
| Large | 0.039 (0.030 to 0.048) | <0.0001 |
| Medium | 0.035 (0.028 to 0.042) | <0.0001 |
| Small | 0.038 (0.030 to 0.045) | <0.0001 |
| Cholesterol | 0.009 (0.007 to 0.011) | <0.0001 |
| Triglycerides | 0.045 (0.036 to 0.054) | <0.0001 |
| IDL particles |  |  |
| Cholesterol | 0.002 (-0.0001 to 0.004) | 0.066 |
| Triglycerides | 0.005 (0.002 to 0.009) | 0.004 |
| LDL particles |  |  |
| Total | 0.003 (-0.007 to 0.013) | 0.61 |
| Large | 0.002 (-0.008 to 0.012) | 0.67 |
| Medium | 0.001 (-0.009 to 0.011) | 0.88 |
| Small | 0.002 (-0.007 to 0.012) | 0.64 |
| Cholesterol | 0.001 (-0.008 to 0.009) | 0.86 |
| Triglycerides | 0.0005 (-0.002 to 0.003) | 0.74 |
| HDL particles |  |  |
| Total | -0.021 (-0.029 to -0.012) | <0.0001 |
| Large | -0.004 (-0.010 to 0.003) | 0.27 |
| Medium | -0.025 (-0.033 to -0.018) | <0.0001 |
| Small | -0.018 (-0.026 to -0.009) | <0.0001 |
| Cholesterol | -0.054 (-0.067 to -0.041) | <0.0001 |
| Triglycerides | 0.004 (0.002 to 0.007) | 0.002 |
| Particle size |  |  |
| VLDL | -0.002 (-0.004 to -0.001) | 0.002 |
| LDL | -0.001 (-0.005 to 0.003) | 0.56 |
| HDL | -0.064 (-0.082 to -0.046) | <0.0001 |
| Conventional lipid measures |  |  |
| Total cholesterol | 0.031 (0.003 to 0.058) | 0.028 |
| LDL cholesterol | 0.040 (0.022 to 0.058) | <0.0001 |
| HDL cholesterol | -0.082 (-0.099 to -0.065) | <0.0001 |
| Remnant cholesterol | 0.044 (0.034 to 0.054) | <0.0001 |
| Non-HDL cholesterol | 0.060 (0.042 to 0.078) | <0.0001 |
| Triglycerides | 0.045 (0.035 to 0.055) | <0.0001 |

Coefficients were calculated for a doubling of the lipid concentration or 0.1 nm increment of the averaged lipoprotein particle size. Abbreviation: HDL, high-density lipoprotein; HDL-C, high-density lipoprotein cholesterol; LDL, low-density lipoprotein; LDL-C, low-density lipoprotein cholesterol; VLDL, very-low-density lipoprotein; WHR, waist-to-hip ratio.

## Table S3. Unadjusted Associations of WHR Obesity with Lipid Parameters

|  | **WHR** | |
| --- | --- | --- |
|  | **Unadjusted OR** | **P** |
| VLDL particles |  |  |
| Total | 2.67 (2.07-3.44) | <0.0001 |
| Large | 2.50 (1.89-3.31) | <0.0001 |
| Medium | 2.40 (1.90-3.03) | <0.0001 |
| Small | 2.69 (2.09-3.47) | <0.0001 |
| Cholesterol | 1.20 (1.13-1.28) | <0.0001 |
| Triglycerides | 3.23 (2.39-4.36) | <0.0001 |
| IDL particles |  |  |
| Cholesterol | 1.09 (1.03-1.16) | 0.002 |
| Triglycerides | 1.21 (1.10-1.33) | <0.0001 |
| LDL particles |  |  |
| Total | 1.17 (0.92-1.49) | 0.21 |
| Large | 1.18 (0.93-1.50) | 0.16 |
| Medium | 1.10 (0.87-1.38) | 0.44 |
| Small | 1.14 (0.91-1.44) | 0.26 |
| Cholesterol | 1.12 (0.91-1.38) | 0.30 |
| Triglycerides | 1.03 (0.96-1.10) | 0.45 |
| HDL particles |  |  |
| Total | 0.77 (0.60-0.98) | 0.031 |
| Large | 1.06 (0.91-1.24) | 0.43 |
| Medium | 0.65 (0.51-0.83) | 0.0006 |
| Small | 0.83 (0.66-1.04) | 0.11 |
| Cholesterol | 0.38 (0.26-0.56) | <0.0001 |
| Triglycerides | 1.14 (1.07-1.21) | <0.0001 |
| Particle size |  |  |
| VLDL | 0.94 (0.90-0.97) | 0.0001 |
| LDL | 1.01 (0.92-1.11) | 0.87 |
| HDL | 0.26 (0.15-0.45) | <0.0001 |
| Conventional lipid measures |  |  |
| Total cholesterol | 3.10 (1.58-6.09) | 0.0010 |
| LDL cholesterol | 2.93 (1.84-4.66) | <0.0001 |
| HDL cholesterol | 0.19 (0.11-0.31) | <0.0001 |
| Remnant cholesterol | 3.08 (2.24-4.25) | <0.0001 |
| Non-HDL cholesterol | 4.86 (2.92-8.08) | <0.0001 |
| Triglycerides | 3.08 (2.23-4.23) | <0.0001 |

ORs and 95% confidence intervals were calculated for a doubling of the lipid concentration or 0.1 nm increment of the averaged lipoprotein particle size. WHR obesity was defined as WHR ≥ 0.85 for women, 0.9 for men. Abbreviation: HDL, high-density lipoprotein; HDL-C, high-density lipoprotein cholesterol; LDL, low-density lipoprotein; LDL-C, low-density lipoprotein cholesterol; VLDL, very-low-density lipoprotein; WHR, waist-to-hip ratio.
